# History and current status of clinical studies using human pluripotent stem cells

**DOI:** 10.1016/j.stemcr.2023.03.005

**Published:** 2023-04-06

**Authors:** Sabine Kobold, Nils Bultjer, Glyn Stacey, Sabine C. Mueller, Andreas Kurtz, Nancy Mah

**Affiliations:** 1 Fraunhofer-Institut für Biomedizinische Technik (IBMT), Joseph-von-Fraunhofer Weg 1, 66280 Sulzbach, Germany; 2 International Stem Cell Banking Initiative, 2 High Street, Barley SG88HZ, UK; 3 Berlin Institute of Health Center for Regenerative Therapies, Augustenburger Platz 1, 13353 Berlin, Germany

## Abstract

The Human Pluripotent Stem Cell Registry established a database of clinical studies using human pluripotent stem cells (PSCs) as starting material for cell therapies. Since 2018, we have observed a switch toward human induced pluripotent stem cells (iPSCs) from human embryonic stem cells. However, rather than using iPSCs for personalized medicines, allogeneic approaches dominate. Most treatments target ophthalmopathies, and genetically modified iPSCs are used to generate tailored cells. We observe a lack of standardization and transparency about the PSCs lines used, characterization of the PSC-derived cells, and the preclinical models and assays applied to show efficacy and safety.

## Teaser

In this perspective, Kobold et al. identify 109 clinical studies using pluripotent stem cells performed in 14 countries targeting 44 diseases by analyzing data from hPSCreg (https://hpscreg.eu/clinical_studies). Persistent lack of standardization and transparency about the source cell lines, characterization of the therapeutic product, applied preclinical models, and efficacy and safety assays are mounting obstacles to clinical study development and reproducibility.

## Introduction

**Figure 1:**
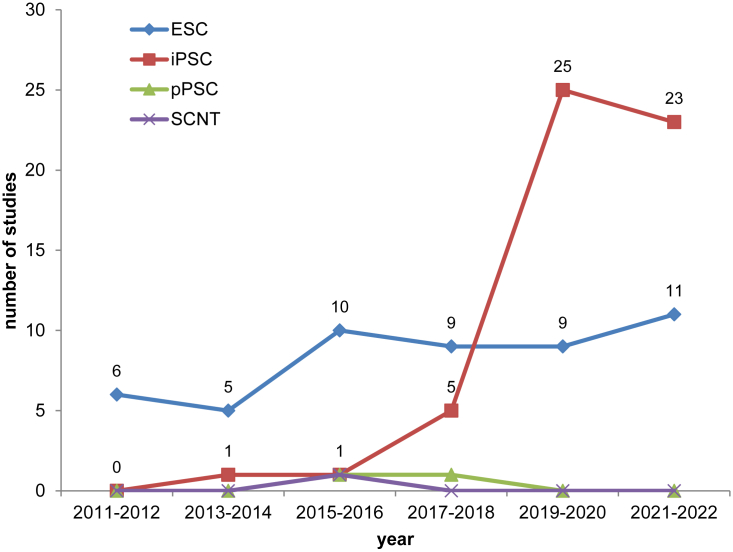
Temporal distribution of studies using ESCs, iPSCs, somatic cell nuclear transfer (SCNT) cells, and parthenotic PSCs (pPSCs) with start dates within the period of January 1, 2011–December 13, 2022 Data are aggregated into 2-year bins.

Clinical studies using human pluripotent stem cells (PSCs) as starting material are promising therapeutic options. For efficient progress in the field at this early stage, it is therefore important to have access to information on ongoing clinical studies, transparency about cell sources, derived cells as a final product, relevant efficacy and safety assays, and outcomes. Because this information is difficult to obtain, a clinical study database dedicated to the use of human PSCs as starting material for cell therapies has been established (Kobold et al., 2020). It is publicly accessible through the Human Pluripotent Stem Cell Registry portal (https://hpscreg.eu; Seltmann et al., 2016; Mah et al., 2020; Kurtz et al., 2022). The clinical study database is continuously updated by keyword searches and manual screening of public resources and includes clinical studies listed at ClinicalTrials.gov (clinicaltrials.gov), the International Clinical Trials Registry Platform (ICTRP) maintained by the World Health Organization (WHO), and multiple other sources (for methods, see Kobold et al., 2020). The database currently lists 109 clinical studies based on human PSC lines (Figure 1), which we further analyzed with a focus on the last 10 years (2012–2022). Furthermore, gaps in available information and data are highlighted, and measures are proposed to improve sharing and access of data related to PSC-based clinical studies to improve reproducibility and reduce regulatory risks.

Our data confirm a strong trend toward studies using human induced PSCs (iPSCs), while human embryonic stem cells (ESCs) continue to be used in a significant portion of roughly 50% of all studies. While the majority of studies up to 2017 utilized ESCs, since 2018, most studies were based on hiPSC-derived products. There is a remarkable drop of clinical studies initiated in 2022 compared with the previous two periods, which may reflect the impact of coronavirus disease 2019 (COVID-19).

### Traceability of the source PSC lines employed in PSC-based therapies

The Human Pluripotent Stem Cell Registry (hPSCreg; https://hpscreg.eu) provides a resource for available iPSC and ESC lines and certifies their identity, ethical provenance, and biological properties, among other related information. Together with its clinical study database, a resource linking information on PSC lines and clinical trials is available at hPSCreg. However, most clinical trials using iPSCs do not publicly disclose the PSC lines used or register these lines in hPSCreg. In fact, we are only able to trace the source PSC cell lines to 11 ESC lines and 1 iPSC line, of which 10 and 0 lines, respectively, are registered in hPSCreg (Table S1).

### Who carries out clinical studies and where

The studies are conducted in only 14 countries (Figure S1), with only 3 newcomer countries (Germany, Iran, and Sweden) hosting trials in the last 5 years. This is indicative of the fact that developing and initiating clinical studies in this field requires considerable time and financial and other resources, including qualified personnel, logistics considerations, and availability of cell lines intended for clinical use (LIFCUs). The pioneering countries overlap with the same countries that perform the most cell and gene therapy studies, which also showed a delayed spread to developing regions (Ginn et al., 2018).

**Figure 2:**
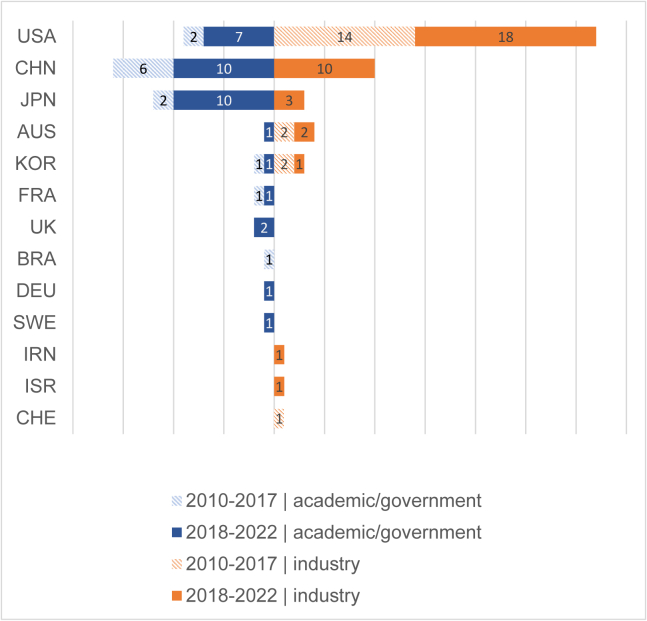
Sponsor type per country (ISO 3166-1 three-letter codes)

The sponsors of clinical studies are the legal entities responsible for initiation, management, and/or financial support of a study (International Conference on Harmonization Guideline for Good Clinical Practice E6(R2); https://www.ema.europa.eu/en/documents/scientific-guideline/ich-guideline-good-clinical-practice-e6r2-step-5_en.pdf). The name of each clinical trial sponsor is a required data element in the ICTRP standards for clinical trial registries who wish to be included in the ICTRP registry network (International Standards for Clinical Trial Registries v.3.0; Geneva, Switzerland, WHO, 2018; license CC BY-NC-SA 3.0 IGO (Intergovernmental Organization)). In most cases (102 of 109 studies), the sponsor can be identified from the clinical study registry record. Within the last 5 years, industry has sponsored more clinical studies since the first PSC-derived cell therapy study was started in 2010 (Figure 2). Industry-sponsored studies have almost doubled in the last 5 years compared with the previous time period (2010–2017), and academically or government-sponsored trials have almost tripled in the same time frame.

There are clear differences between countries. While in the United States, the number of industry-sponsored trials outweighs academically/government-sponsored trials (32 and 9, respectively) and are strongly represented even before 2018, Japan and China have only within the last 5 years started trials with industry sponsors, and the bulk of their trials remains predominantly academically/government sponsored as a result of strong national funding programs (Yuan et al., 2012; Enosawa, 2022; Li, 2022). Only a handful of clinical trials (5, in the United States, Japan, and China) are investigator-initiated trials. Big pharma is not the major player in sponsoring the studies; worldwide, the vast majority (70%) of industry-sponsored trials are conducted by smaller biotech companies.

### Cell types and their target indications for PSC-based therapies

**Figure 3:**
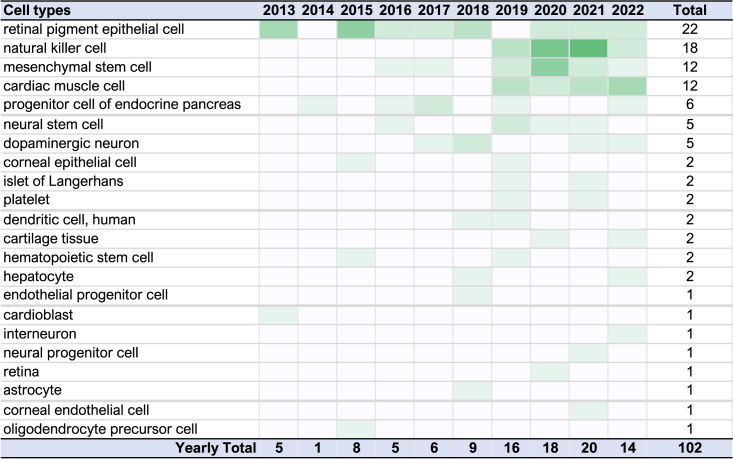
Heatmap showing frequencies of PSC-derived cell types used in clinical studies for the last decade

Within the last decade, the number of PSC-derived cell types used for therapies has grown from 3 to 22 cell types, encompassing 44 target indications/diseases (Table S2). The most frequent therapeutic cell type focused on in the last 10 years is the retinal pigment epithelial cell, and PSC derivatives of this type have been used to treat degenerative eye diseases in 22 clinical trials (Figure 3). In second place, natural killer (NK) cell-like derivatives are involved in 18 clinical studies, all of which have been started in the last 5 years. Over 75% of these NK studies have been initiated by a single company. The engineered specificity of the chimeric antigen receptor (CAR)-T cells endows these therapies with the ability to target liquid or solid tumors. Tissue-specific stem cells (TSCs) or mesenchymal stem cell-like and cardiomyocyte-like cells are used in 12 studies for each cell type. PSC-derived TSC products are undergoing a kind of renaissance in the PSC therapy field because of the immunomodulatory properties of native TSC cell-based therapies and are indicated for diverse disease targets (Table S3). Cardiomyocyte-like cells are primarily used to treat heart failure. The remainder of the cell types have fewer than three indications per cell type, which include neuronal cell types for spinal cord injury or Parkinson’s disease, pancreatic cells for type 1 diabetes, and hematopoetic stem cells for β-thalassemia, to name but a few.

The multiplicity of studies employing a single cell type also reflects different clinical study phases of the same cell product (such as follow-up trials or trials conducted in conjunction with other established drugs) or clinical application of the cells to diseases where cell degeneration is a common denominator. Another important additional consideration is clinical testing of any medical devices that have been developed to deliver the cell product to the appropriate site in the patient (Creasey et al., 2019). For example, a transplant delivery system to deliver oligodendrocyte progenitor cells to the spinal cord is being pursued for the cell product OPC1 (clinicaltrials.gov: NCT02302157).

Disease targets for PSC-based therapies have broadened moderately (Table S2). While 16 of 30 (∼53%) of clinical studies initiated in the period from 2011–2017 target eye diseases, 66 of 78 (∼85%) of studies target other diseases in studies initiated between 2018 and 2022. The recruitment period for the clinical studies on eye diseases varied between 2 and 7 years, and 15 studies were to be completed by 2022; however, published data do not yet allow meta-analysis of outcomes. Of the 109 clinical studies recorded in the clinical study database in hPSCreg, only a fraction have associated scientific publications. Altogether, there are 31 linked publications for 18 studies (www.hpscreg.eu/browse/trials). For these 31 publications, 5 include preclinical work, 1 reports only preclinical data, 5 include manufacturing protocols and cell quality assessment parameters, and 30 report clinical study design and outcomes. Reports on termination, withdrawal, or failure of clinical studies are not published and, unfortunately, only in a few cases recorded in public registries. One could assume that those 17 studies that published outcome results are completed, and, thus, that, of the 109 recorded clinical studies, at most 93 are currently active.

Study duration, as defined by time between study start and study, is available for 90 of 109 studies and varies between 1 and 19 years (median, 7 years; average, 6.5 years). The time frame from start to completion may serve as a surrogate indicator for follow-up duration. No correlation was found between the duration of study, national regulatory framework, year when the study started, or disease category.

## Discussion

### Data requirements for tracking the progress of PSC-derived stem cell therapies

The sourced database hosted by hPSCreg uses a combination of search tools to gather information on worldwide clinical studies using PSCs as source material. The data are manually curated before being entered into the clinical study database and made publicly accessible at the hPSCreg portal (https://hpscreg.eu; Guhr et al., 2018; Kobold et al., 2020). Study investigators are encouraged to enter clinical studies and to update, edit, and enrich the initial data provided by the portal. Key clinical study data that are frequently not publicly available and should be provided by investigators include the identity of the PSC line used and information regarding characterization and quality control (QC). When a PSC line is registered in hPSCreg, a unique, persistent identifier is assigned to it. Interoperability via this identifier ensures linking the cell line not only to the clinical study but also to all information related to the cell line in published resources. This also applies to the final therapeutic cell preparation, where characterization, QC, and assays for efficacy and safety analysis are ideally made available. Such external data can be directly entered or linked from any source where it is deposited, generating a federated informative environment for PSC-based clinical studies. To further enrich the clinical study information, published reports or patents can also be linked.

Public clinical study registries, resources, and trial publications often do not use the same standards for reporting, which makes comparisons of data across the field and understanding potential causes of trial failure difficult. The hPSCreg database orients its reporting requirements on the WHO data fields to ease cross-linking of information, adhering to the International Committee of Medical Journal Editors (ICMJE) recommendations (https://icmje.org/recommendations/). Moreover, information on cells used (i.e., a published source cell line registered in hPSCreg or another registry) is only occasionally available. Furthermore, although employed in hPSCreg, standardized ontologies, taxonomies, or terms to describe cell- or disease-specific data are rarely used in other clinical trial registries. Access to the data, comparability, and searchability require further use of standard terms and taxonomies; for example, for therapeutic cell type, target clinical feature or disease, or experimental procedures.

### Current levels of accessibility to key data stunts progress in PSC-derived clinical applications

The importance of data sharing is increasingly recognized but not strictly enforced. The clinicaltrial.gov registry (https://beta.clinicaltrials.gov/) encourages sharing of output data and study-related publications, albeit on a voluntary basis. For example, the ICMJE introduced a requirement for authors to report how individual patient data (IPD) will be shared when publishing clinical trial results (Statham et al., 2020). A recent study showed that a data sharing statement was largely missing in study records analyzed in ICTRP (132,545 studies from January 2019–December 2020) (Merson et al., 2022). Output reporting is a prerequisite to making meaningful associations with preclinical data, potency, and safety assays, but such interpretation is currently hindered by the lack of public information and its poorly standardized presentation. There is no report of termination of a clinical study of allogenic PSC-derived product application. However, one case has been reported where treatment was not performed based on safety concerns. Here, a genetic change of unknown consequences was detected in the autologous iPSCs and the derived retinal pigment epithelial cells intended for a patient with macular degeneration, triggering precautionary withdrawal by the investigators. The treatment results of the first patients enrolled in this study were also reported (Mandai et al., 2017; Takagi et al., 2019). It must be noted that information on termination, suspension, or the reasons for these are rarely available in public resources. The same applies to study-related information on lack of efficacy and preclinical assays used to assess efficacy or safety. Indeed, compliance with reporting clinical study results within 12 months of the end of the trial is poor (Goldacre et al., 2018; DeVito et al., 2020).

Information on the identity, source, and QC protocols of source PSCs is not transparent for the overwhelming majority of PSC-based clinical studies. Similarly, access to information on the end products, such as characterization details and assays to determine safety and potency, are only in rare cases publicly accessible. These data are essential for regulatory approval; thus, we believe that sharing such information may help to establish standards, which may then help regulatory processes on a global scale. The lack of available information on assays used to elucidate the safety and efficacy of source and product cells poses risks for reproducibility and increases global costs, not the least because each regulatory authority may require assay requalification. A clinical study output analysis together with data on preclinical assessment protocols will eventually help to reduce these risks and promote use of cross-validated potency and safety assessment protocols.

Besides access to data, the availability of LIFCUs is another key factor for initiating clinical studies. Because most studies use allogeneic procedures and do not match HLA types, established LIFCUs could, in principle, be used in multiple clinical studies. In fact, the great advantage of iPSCs to allow autologous cell therapies is only exploited in 10 of 109 studies. This may be because of inherent manufacturing risks with regard to genetic stability and current manufacturing costs (Lopes et al., 2018; Madrid et al., 2021). Use of donor HLA-matched PSC lines is an alternative strategy requiring access to haplotyped lines suitable for a broad donor population (Nakatsuji et al., 2008). However, this approach may require additional regulatory oversight or even a new clinical trial for each different cell line used (www.iscbi.org/publications). Relevant resources are now emerging, such as the European Union (EU)-funded COST (Cooperation in Science & Technology) innovation network “Generation of Human Induced Pluripotent Stem Cells From Haplo-Selected Cord Blood Samples” (HAPLO-iPS; CA21151); however, haplotyped iPSC lines have not yet been broadly exploited for clinical studies (Sullivan et al., 2020). Genetically engineered hypoimmunogenic iPSC are being used in one clinical study (ClinicalTrials.gov: NCT05210530; Philippidis, 2022). In the common allogeneic settings, there is no advantage obtained when using iPSCs compared with ESCs. In contrast, ESCs do not require genetic reprogramming, and, therefore, the initial manufacturing process is less complex with fewer cell manipulations. Moreover, licensing and, thus, the commercial freedom to operate are easier to manage than for iPSCs. However, use of ESCs is restricted in some countries because of regulatory restrictions based on ethical controversies (Of stem cells and ethics, 2017).

Clinical applications of PSC-derived cells are only being pursued in 14 countries worldwide, with little or no activity in Africa, Eastern Europe, South America, and Asia (outside of China, Japan, and Korea). These trends confirm an enormous imbalance of global resources and likely will perpetuate unequal access to therapeutic benefits, which, of course, is not restricted to advanced therapies (Cornetta et al., 2022; Muigai, 2022). Measures to minimize this access gap should be developed already at early stages on a societal level. Different regulatory and legal frameworks may additionally hinder or delay cross-border spread of clinical studies. However, the effect can only be assumed because there is no coherent information on the time needed from initial conception to starting the clinical trial, which may be up to 18 years. That the latter may be relevant could be deduced from the regulatory changes implemented, for example, in Japan (SAKIGAKE), the United States (Regenerative Medicine Advanced Therapy [RMAT] designation), and now in the EU (Priority Medicines [PRIME]) to streamline the process for approval of cell therapies (Pimenta et al., 2021). Furthermore, as an example, the strict German ESC regulations prevent development of ESC-based therapies because of existing marketing risks. As a novel type of drug product, cell-based therapies have no predestined development path—each product requires a series of consultations with the relevant regulatory bodies for its progress through clinical trials or special exemptions. Unlike traditional drug development, “the product is the process,” but measures to harmonize requirements for reporting LIFCUs and the derived end products may be possible and support clinical study development and reproducibility.

### Conclusions

The number of PSC-based cell clinical studies has rapidly increased in the last 5 years. In the next decade, we expect to see more strategic collaborations within industry as big pharma seeks to acquire the expertise of smaller companies to enter the cell therapy arena. At the same time, smaller companies forge strategic alliances with one another through licensing or collaboration agreements (Ilic and Liovic, 2023). Mergers and acquisitions in the field will also likely increase; examples include Fujifilm/Cellular Dynamics International (2015), Bayer/Blue Rock Therapeutics (2019), Catalent/RheinCell Therapeutics (2021), and Pluristyx/panCELLa/Implant Therapeutics (2022). We anticipate more innovations, such as clinical studies addressing transplant delivery devices and hypoimmune gene-edited lines. Autologous applications may become standard for established applications and dependent on decreasing cost dynamics eventually dominate. With the anticipated growth of clinical studies, improvements in the accessibility of data and comparability of all aspects of clinical studies, from source material to outcome, combined with consistent application of reporting standards, would encourage harmonization on a global level. Such standardization would help significantly to reduce the regulatory and financial burden each single sponsor will have to shoulder and could accelerate acceptability of these novel therapeutics. The full potential of cell-based therapies in all parts of the world can only be realized if regulators, funders, and clinical investigators work together to make the cell-based therapy development process more transparent by sharing critical data required for development of these therapies.

## Author contributions

S.K., collection and assembly of data, data analysis, and generation of figures; N.B., implementation of the database and collection and assembly of data; G.S. and A.K., conception, design, and writing of the manuscript; S.C.M., writing and revision of the manuscript; N.M., data analysis and interpretation, manuscript writing, and generation of figures and tables.

## Supplemental information

Document S1. Figure S1 and Tables S1–S3

Document S2. Article plus supplemental information
