# Supplementary material for: History and current status of clinical studies using human pluripotent stem cells
Source: Stem Cell Reports. 2023 Apr 6;18(8):1592–8. doi: 10.1016/j.stemcr.2023.03.005 (PMC10444540; doi:10.1016/j.stemcr.2023.03.005)
Supplement: Document S1. Figure S1 and Tables S1–S3 [file mmc1.pdf]

**Stem Cell Reports, Volume *18***

## Supplemental Information

# History and current status of clinical studies using human pluripotent stem cells

**Sabine Kobold, Nils Bultjer, Glyn Stacey, Sabine C. Mueller, Andreas Kurtz, and Nancy Mah**

**Supplementary Information for:**

# History and current status of clinical studies using human pluripotent stem cells

Sabine Kobold[1], Nils Bultjer[1], Glyn Stacey[2], Sabine C. Mueller[1], Andreas Kurtz[1,3], Nancy Mah[1]

[1]Fraunhofer-Institut für Biomedizinische Technik (IBMT), Joseph-von-Fraunhofer Weg 1, 66280 Sulzbach, Germany
[2] International Stem Cell Banking Initiative, 2 High Street, Barley SG88HZ, UK
[3]Berlin Institute of Health Center for Regenerative Therapies, Augustenburger Platz 1, 13353 Berlin, Germany

## List of Supplementary Information

**Supplementary Figure S1.** World map of clinical study sites, related to section "Where and who carries out clinical studies".

**Supplementary Table S1**. PSC lines used as starting materials for the production of cell-based therapeutics, related to section "Traceability of the source PSC lines employed in PSC-based therapies".

**Supplementary Table S2**. Target diseases of PSC-derived cell therapies, related to section "Cell types and their target indications for PSC-based therapies".

**Supplementary Table S3.** Cell types used for PSC-derived cell-based therapies (last 10 years) and target indications, related to section "Cell types and their target indications for PSC-based therapies".

**Figure S1.** World map of clinical study sites, related to section “Where and who carries out clinical studies”.

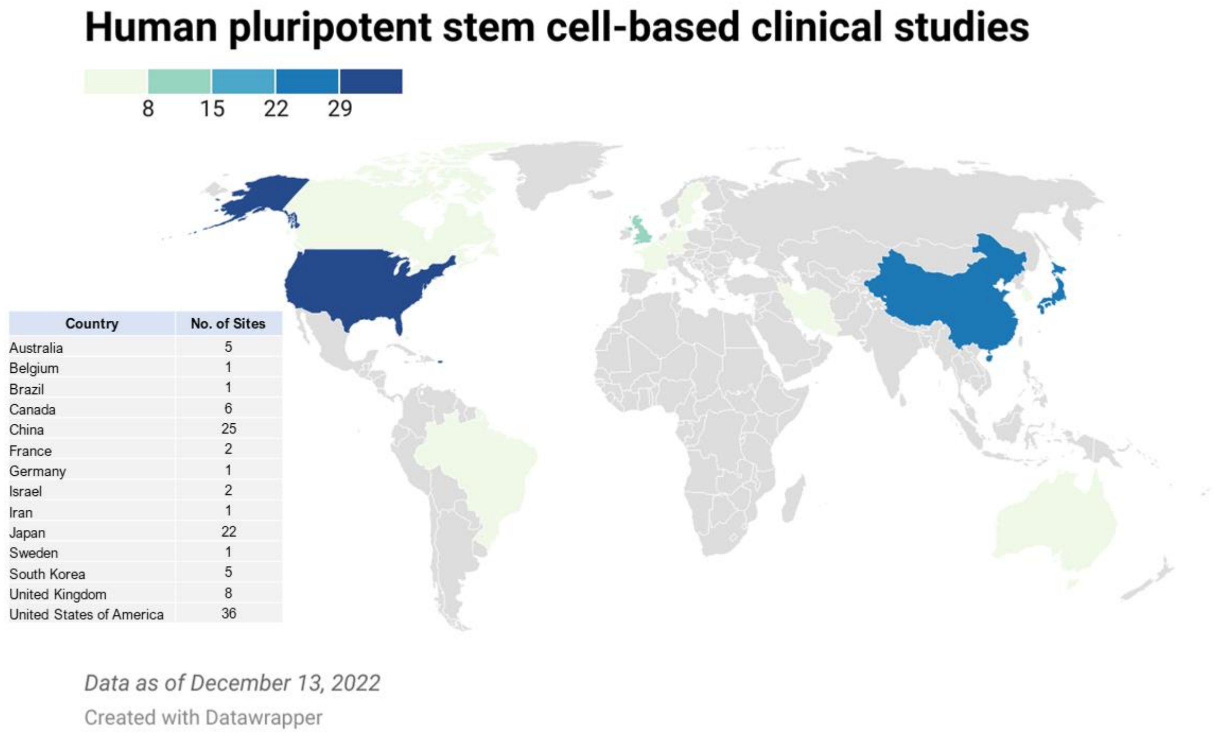


| Country | No. of Sites |
|---|---|
| Australia | 5 |
| Belgium | 1 |
| Brazil | 1 |
| Canada | 6 |
| China | 25 |
| France | 2 |
| Germany | 1 |
| Israel | 2 |
| Iran | 1 |
| Japan | 22 |
| Sweden | 1 |
| South Korea | 5 |
| United Kingdom | 8 |
| United States of America | 36 |

**Table S1.** PSC lines used as starting materials for the production of cell-based therapeutics, related to section "Traceability of the source PSC lines employed in PSC-based therapies".

| PSC line common name | hPSCreg® cell line ID | Year of First Publication of the Cell Line | No. of Clinical Trials |
|---|---|---|---|
| Human embryonic stem cells | | | |
| MA09 | ACTe002-A | 2006 | 13 |
| CyT49 | VCYTe001-A | 2006 | 6 |
| H1 | WAe001-A | 1998 | 3 |
| H9 | WAe009-A | 1998 | 2 |
| Shef-1 | AXORe001-A | 2004 | 2 |
| HAD-C 100 | HADe008-A | 2012 | 1 |
| HAD-C 102 | HADe007-A | 2012 | 1 |
| I6 | TECHe003-A | 2002 | 1 |
| RC17 | RCe021-A | 2016 | 1 |
| RC9 | RCe013-A | 2012 | 1 |
| Q-CTS-hESC-2 | Not registered | 2016 | 1 |
| Unknown ESC | | | 19 |
| Human induced pluripotent stem cells | | | |
| QHJI | Not registered | 2019 | 3 |
| Unknown iPSC | | | 51 |

**Table S2.** Target diseases of PSC-derived cell therapies, related to section “Cell types and their target indications for PSC-based therapies”.

| Disease (ICD-10 classification) | Cellular Origin of Cell Product hESC | hiPSC | hpSC | hSCNT | Total |
|---|---|---|---|---|---|
| **Diseases of the eye and adnexa** | **24** | **9** | **0** | **1** | **34** |
| Degeneration of macula and posterior pole: Senile macular degeneration, dry AMD | 11 | 3 | | 1 | |
| Other retinal disorders | 4 | 2 | | | |
| Hereditary retinal dystrophy: Stargardt disease | 5 | | | | |
| Hereditary retinal dystrophy: retinitis pigmentosa | 2 | 1 | | | |
| Hereditary retinal dystrophy | 1 | | | | |
| Bullous keratopathy | | 1 | | | |
| Other specified disorders of cornea | | 1 | | | |
| Degeneration of macula and posterior pole: wet age-related macular degeneration | | 1 | | | |
| Myopia | 1 | | | | |
| **Neoplasms** | **1** | **17** | **0** | **0** | **18** |
| Malignant neoplasms | | 5 | | | |
| Myeloid leukaemia | | 3 | | | |
| Other and unspecified types of non-Hodgkin lymphoma | | 2 | | | |
| Chronic lymphocytic leukaemia of B-cell type | | 2 | | | |
| Malignant neoplasm of ovary | | 2 | | | |
| Head, face and neck | | 1 | | | |
| Malignant neoplasm of bronchus and lung | 1 | | | | |
| Malignant neoplasm of breast | | 1 | | | |
| Multiple myeloma | | 1 | | | |
| **Diseases of the circulatory system** | **3** | **13** | **0** | **0** | **16** |
| Ischaemic cardiomyopathy | | 7 | | | |
| Cerebral infarction, unspecified | 1 | 2 | | | |
| Heart failure, unspecified | | 2 | | | |
| Left ventricular failure | 1 | | | | |
| Ischaemic heart diseases | 1 | | | | |
| Congestive heart failure | | 1 | | | |
| Dilated cardiomyopathy | | 1 | | | |
| **Diseases of the nervous system** | **5** | **3** | **2** | **0** | **10** |
| Parkinson disease | 2 | 3 | 2 | | |
| Multiple sclerosis | 1 | | | | |
| Epilepsy | 1 | | | | |
| Motor neuron disease | 1 | | | | |

| Disease (ICD-10 classification) | Cellular Origin of Cell Product hESC | hiPSC | hpSC | hSCNT | Total |
|---|---|---|---|---|---|
| **Endocrine, nutritional and metabolic diseases** | **9** | **2** | **0** | **0** | **11** |
| Unspecified diabetes mellitus with multiple complications with diabetic foot syndrome, controlled | | 1 | | | |
| Type 1 diabetes mellitus | 7 | 1 | | | |
| Disorders of urea cycle metabolism | 1 | | | | |
| Primary ovarian failure | 1 | | | | |
| **Injury, poisoning and certain other consequences of external causes** | **3** | **4** | **0** | **0** | **7** |
| Injury of spinal cord, level unspecified | 3 | 1 | | | |
| Tear of articular cartilage of knee, current | | 2 | | | |
| Bone-marrow transplant rejection | | 1 | | | |
| **Diseases of the blood and blood-forming organs and certain disorders involving the immune mechanism** | **0** | **4** | **0** | **0** | **4** |
| Beta thalassaemia | | 2 | | | |
| Thrombocytopenia, unspecified | | 1 | | | |
| Other aplastic anaemias | | 1 | | | |
| **Provisional assignment of new diseases of uncertain etiology or emergency use** | **2** | **2** | **0** | **0** | **4** |
| COVID-19 | 2 | 2 | | | |
| **Diseases of the genitourinary system** | **2** | **0** | **0** | **0** | **2** |
| Intrauterine synechiae | 1 | | | | |
| Interstitial cystitis (chronic) | 1 | | | | |
| **Arthropathies** | **0** | **1** | **0** | **0** | **1** |
| Osteoarthritis of knee, unspecified | | 1 | | | |
| **Diseases of the digestive system** | **1** | **0** | **0** | **0** | **1** |
| Acute and subacute hepatic failure | 1 | | | | |
| **Diseases of the musculoskeletal system and connective tissue** | **1** | **0** | **0** | **0** | **1** |
| Derangement of meniscus due to old tear or injury | 1 | | | | |
| **Totals** | **51** | **55** | **2** | **1** | **109** |

**Table S3.** Cell types used for PSC-derived cell-based therapies (last 10 years) and target indications, related to section “Cell types and their target indications for PSC-based therapies”.

| Cell types used for therapy | 2013 | 2014 | 2015 | 2016 | 2017 | 2018 | 2019 | 2020 | 2021 | 2022 | Total |
|---|---|---|---|---|---|---|---|---|---|---|---|
| **retinal pigment epithelial cell** | **4** | | **5** | **2** | **2** | **3** | | **2** | **2** | **2** | **22** |
| age related macular degeneration | 2 | | 2 | 2 | 2 | 2 | | 1 | 1 | 1 | 13 |
| dystrophies primarily involving the retinal pigment epithelium | | | | | | | | | 1 | | 1 |
| macular degeneration | | | 2 | | | 1 | | | | | 3 |
| myopic macular degeneration | 1 | | | | | | | | | | 1 |
| retinitis pigmentosa | | | 1 | | | | | 1 | | | 2 |
| RPE impaired disease | | | | | | | | | | 1 | 1 |
| Stargardt disease | 1 | | | | | | | | | | 1 |
| **natural killer cell** | | | | | | | **3** | **6** | **7** | **2** | **18** |
| acute myeloid leukemia | | | | | | | 1 | 1 | 1 | | 3 |
| advanced malignant solid neoplasm | | | | | | | 2 | 1 | 1 | 1 | 5 |
| B-Cell malignant neoplasm | | | | | | | | | | 1 | 1 |
| breast cancer | | | | | | | | | 1 | | 1 |
| chronic lymphocytic leukemia | | | | | | | | 1 | 1 | | 2 |
| COVID-19 | | | | | | | | 1 | | | 1 |
| head and neck cancer | | | | | | | | 1 | | | 1 |
| multiple myeloma | | | | | | | | | 1 | | 1 |
| non-Hodgkin lymphoma | | | | | | | | 1 | | | 1 |
| ovarian cancer | | | | | | | | | 2 | | 2 |
| **mesenchymal stem cell** | | | | **1** | **1** | | **2** | **5** | **2** | **1** | **12** |
| adhesions of uterus | | | | | | | | 1 | | | 1 |
| age related macular degeneration | | | | 1 | | | | | | | 1 |
| COVID-19 | | | | | | | | 3 | | | 3 |
| diabetic foot ulcer | | | | | | | | | | 1 | 1 |
| graft versus host disease | | | | | 1 | | | | | | 1 |
| interstitial cystitis | | | | | | | | 1 | | | 1 |
| meniscus | | | | | | | 1 | | | | 1 |
| multiple sclerosis | | | | | | | | | 1 | | 1 |
| osteoarthritis | | | | | | | | | 1 | | 1 |
| primary ovarian insufficiency | | | | | | | 1 | | | | 1 |
| **cardiac muscle cell** | | | | | | | **3** | **2** | **3** | **4** | **12** |
| cardiomyopathy | | | | | | | 1 | 1 | | 1 | 3 |
| congestive heart failure | | | | | | | | | 1 | | 1 |
| heart failure | | | | | | | 1 | 1 | 2 | 2 | 6 |
| ischemic heart disease | | | | | | | 1 | | | 1 | 2 |
| **progenitor cell of endocrine pancreas** | | **1** | | **1** | **2** | | **1** | | | **1** | **6** |
| type 1 diabetes mellitus | | 1 | | 1 | 2 | | 1 | | | 1 | 6 |
| **neural stem cell** | | | | **1** | | | **2** | **1** | **1** | | **5** |
| ischemic cerebrovascular accident | | | | | | | 1 | | 1 | | 2 |
| Parkinson's disease | | | | 1 | | | 1 | | | | 2 |

| Cell types used for therapy | 2013 | 2014 | 2015 | 2016 | 2017 | 2018 | 2019 | 2020 | 2021 | 2022 | Total |
|---|---|---|---|---|---|---|---|---|---|---|---|
| spinal cord injury | | | | | | | | 1 | | | 1 |
| **dopaminergic neuron** | | | | | **1** | **2** | | | **1** | **1** | **5** |
| Parkinson's disease | | | | | 1 | 2 | | | 1 | 1 | 5 |
| **corneal epithelial cell** | | | **1** | | | | **1** | | | | **2** |
| limbal stem cell deficiency | | | 1 | | | | 1 | | | | 2 |
| **islet of Langerhans** | | | | | | | **1** | | **1** | | **2** |
| type 1 diabetes mellitus | | | | | | | 1 | | 1 | | 2 |
| **platelet** | | | | | | | **1** | | **1** | | **2** |
| aplastic anemia | | | | | | | 1 | | | | 1 |
| thrombocytopenia | | | | | | | | | 1 | | 1 |
| **dendritic cell, human** | | | | | | **1** | **1** | | | | **2** |
| lung non-small cell carcinoma | | | | | | 1 | | | | | 1 |
| retinitis pigmentosa | | | | | | | 1 | | | | 1 |
| **cartilage tissue** | | | | | | | | **1** | | **1** | **2** |
| articular cartilage disease | | | | | | | | 1 | | 1 | 2 |
| **hematopoietic stem cell** | | | **1** | | | | **1** | | | | **2** |
| beta thalassemia | | | 1 | | | | 1 | | | | 2 |
| **hepatocyte** | | | | | | **1** | | | | **1** | **2** |
| acute liver failure | | | | | | | | | | 1 | 1 |
| urea cycle disorder | | | | | | 1 | | | | | 1 |
| **endothelial progenitor cell** | | | | | | **1** | | | | | **1** |
| ischemic cerebrovascular accident | | | | | | 1 | | | | | 1 |
| **cardioblast** | **1** | | | | | | | | | | **1** |
| ischemic heart disease | 1 | | | | | | | | | | 1 |
| **interneuron** | | | | | | | | | | **1** | **1** |
| epilepsy | | | | | | | | | | 1 | 1 |
| **neural progenitor cell** | | | | | | | | | **1** | | **1** |
| spinal cord injury | | | | | | | | | 1 | | 1 |
| **retina** | | | | | | | | **1** | | | **1** |
| retinitis pigmentosa | | | | | | | | 1 | | | 1 |
| **astrocyte** | | | | | | **1** | | | | | **1** |
| amyotrophic lateral sclerosis | | | | | | 1 | | | | | 1 |
| **corneal endothelial cell** | | | | | | | | | **1** | | **1** |
| bullous keratopathy | | | | | | | | | 1 | | 1 |
| **oligodendrocyte precursor cell** | | | **1** | | | | | | | | **1** |
| spinal cord injury | | | 1 | | | | | | | | 1 |
| Grand Total | 5 | 1 | 8 | 5 | 6 | 9 | 16 | 18 | 20 | 14 | 102 |
